# Role of Alcohol Metabolism in Non-Alcoholic Steatohepatitis

**DOI:** 10.1371/journal.pone.0009570

**Published:** 2010-03-08

**Authors:** Susan S. Baker, Robert D. Baker, Wensheng Liu, Norma J. Nowak, Lixin Zhu

**Affiliations:** 1 Digestive Diseases and Nutrition Center, Department of Pediatrics, The State University of New York, Buffalo, New York, United States of America; 2 Department of Biochemistry and the New York State Center of Excellence in Bioinformatics and Life Sciences, The State University of New York at Buffalo, Buffalo, New York, United States of America; 3 Microarray and Genomics Facility, Roswell Park Cancer Institute, Buffalo, New York, United States of America; Copenhagen University Hospital, Denmark

## Abstract

**Background:**

Non-alcoholic steatohepatitis (NASH) is a serious form of non-alcoholic fatty liver disease (NAFLD), associated with obesity and insulin resistance. Previous studies suggested that intestinal bacteria produced more alcohol in obese mice than lean animals.

**Methodology/Principal Findings:**

To investigate whether alcohol is involved in the pathogenesis of NASH, the expression of inflammation, fibrosis and alcohol metabolism related genes in the liver tissues of NASH patients and normal controls (NCs) were examined by microarray (NASH, n = 7; NC, n = 4) and quantitative real-time PCR (NASH, n = 6; NC, n = 6). Genes related to liver inflammation and fibrosis were found to be elevated in NASH livers compared to normal livers. The most striking finding is the increased gene transcription of alcohol dehydrogenase (ADH) genes, genes for catalase and cytochrome P450 2E1, and aldehyde dehydrogenase genes. Immunoblot analysis confirmed the increased expression of ADH1 and ADH4 in NASH livers (NASH, n = 9; NC, n = 4).

**Conclusions/Significance:**

The augmented activity of all the available genes of the pathways for alcohol catabolism suggest that 1) alcohol concentration was elevated in the circulation of NASH patients; 2) there was a high priority for the NASH livers to scavenge alcohol from the circulation. Our data is the first human evidence that suggests alcohol may contribute to the development of NAFLD.

## Introduction

Non-alcoholic steatohepatitis (NASH) is a serious form of non-alcoholic fatty liver disease (NAFLD), characterized by hepatic steatosis associated with evidence of inflammation and variable degrees of fibrosis [1]. The prevalence of NASH is increasing with the ever-growing problem of obesity in the general population. Up to 20% of the population may have fatty liver, and up to 3% may have NASH as estimated in 2004 [2].

According to the current “two-hit” hypothesis, NASH patients first develop fatty liver before a second hit initiates inflammation in the liver [3]. Studies with NASH patients and various animal models in the last decade have revealed insights into liver fat infiltration, damage, inflammation and fibrosis. Insulin resistance (IR) seems to be a major reason for fat deposition in liver by way of uncontrolled lipolysis in adipose tissue leading to increased levels of circulating free fatty acids [4]. Measurement with stable isotopes in NAFLD patients indicated that free fatty acids are the major source for fat accumulation in liver. De novo lipogenesis and dietary fatty acids are also significant sources of fat [5]. Other studies suggested that insufficient fatty acid utilization could also cause fat desposition in hepatocytes. Peroxisome proliferator-activated receptors are transcriptional factors regulating lipid metabolism in liver and therefore believed to play a central role in hepatic fat accumulation [6]. Lipotoxicity may cause mitochondrial dysfunction and subsequent liver damage and trigger inflammation [7]. Oxidative stress is possibly a potent thrust in this process and in subsequent activation of hepatic stellate cells [8].

Despite these efforts, the exact mechanism of the development of NASH is unclear. And it remains a puzzle that NASH and alcoholic steatohepatitis share many histological features [9]. Both NASH and alcoholic steatohepatitis patients exhibit macrovesicular and microvesicular fat in hepatocytes. The number and size of Mallory bodies, and the pattern of pericellular fibrosis are also indistinguishable between two disease groups. Previous studies suggested that intestinal bacteria produced more alcohol in obese mice than lean animals [10]. Therefore, we hypothesize that alcohol is involved in the pathogenesis of NASH.

In this report, gene expression in NASH livers was examined by a whole-genome DNA microarray and quantitative real-time PCR (qRT-PCR). We found that all the available genes of the pathways for alcohol catabolism exhibited elevated expression levels in NASH livers, among which the elevated protein levels of ADH1 and ADH4 in NASH livers were confirmed by Western blot analysis. Our results suggest a key role for intestinal alcohol in the pathogenesis of NASH.

## Methods

### Patients

The human studies have been approved by the Institutional Review Board (IRB) of the State University of New York at Buffalo. Only children and adolescents were included in this study to ensure that our patients were not sustained alcohol users. All NASH patients included in this study (Table 1) exhibited significant insulin resistance (IR). IR was calculated based on the “homeostasis model assessment (HOMA)” method [11]. Liver biopsies were obtained, with prior written consent, from parents of patients suspected of having NASH as part of regular medical care. Patients from 7 to 18 years of age signed an assent to the research. Diagnosis of NASH was based on hepatic fat infiltration, inflammation and fibrosis as revealed by liver biopsy, following Kleiner's criteria [12].

**Table 1:** Characteristics of study groups.

	Microarray		Real-time PCR		Western blot	
	NASH	Normal control†	NASH	Normal control†	NASH	Non-NASH control‡
Sex (F ∶ M)	F2 ∶ M5	F1 ∶ M3	F2 ∶ M3	F2 ∶ M4	F4 ∶ M5	F3 ∶ M1
Age (years)	9–16	1–19	9–14	1–9	11–18	4–12
Body Mass Index	35.0±2.9§	19.1±1.6¶	38.4±5.9§	16.5±0.8¶	32.7±1.6§	20.6±2.9¶
Fasting Insulin (mU/ml)	27.0±2.2	NA	31.2±2.3	NA	20.7±5.1	<2
Fasting glucose (mmol/L)	5.0±0.2	NA	5.1±0.3	NA	5.7±0.4	4.7±0.2
IR (HOMA)††	6.0±0.5	NA	7.0±0.5	NA	5.7±1.7	<1

† Normal healthy liver intended for transplantation, no liver disease reported.

‡ Liver biopsies from patients with hepatitis C, autoimmune hepatitis, gall stones or cystic fibrosis, respectively. They were free from steatosis.

§ Patients are all above 95 percentile of the population.

¶ Normal controls are all below 80 pencentile of the population.

†† IR (HOMA) for healthy subjects is around 1.

NA: Not available.

For non-steatosis controls, total RNA was purchased from Admet Technologies (Durham, NC). These samples were derived from liver grafts of normal controls with normal body mass index (Table 1). The ages of these normal controls were similar to our NASH patients. No liver diseases were diagnosed with these normal controls. The healthy status of these livers was ascertained by the lower transcription levels of marker genes for inflammation and fibrosis, as detailed in the Results section. The non-NASH controls used in Western blot analysis were liver biopsies from patients with hepatitis C, autoimmune hepatitis, gall stones or cystic fibrosis, respectively. They were free from steatosis.

### RNA Extraction and Microarray Hybridization

Liver biopsies were stored in RNAlater before total RNA was extracted with RNeasy and treated with RNase-free DNase I set (Qiagen, Valencia, CA). RNA samples obtained from Admet were also treated with RNase free DNase before downstream experiments. Quality of the RNA samples was assured with Bioanalyzer (Agilent Technologies) before downstream biotin-labeling and hybridization to the CodeLink Human Whole Genome Bioarray (GE Health care-Amersham Biosciences, Piscataway, NJ) following the manufacturer's manual. The original microarray data have been uploaded to Gene Expression Omnibus (GEO) website: http://www.ncbi.nlm.nih.gov/geo/index.cgi. All data is MIAME compliant. The accession numbers for NASH liver datasets are GSM435821 to GSM435827, and for normal control datasets: GSM435828, GSM435833 to GSM435835.

### Quantitative RT-PCR

Specific primers (Table 2) were designed with the assistance of Primer 3 [13]. Complementary DNA was synthesized from 0.8 µg of RNA in a volume of 20 µl, using the iScript cDNA synthesis kit (Bio-Rad Laboratories, Hercules, CA). Real-time PCR was performed on an iCycler iQ real-time detection system (Bio-Rad Laboratories, Hercules, CA), using Sybergreen (iQ™ SYBR® Green Supermix; Bio-Rad Laboratories, Hercules, CA) for real-time monitoring of the PCR. GAPD RNA level were tested in parallel with the genes of interest. The presence of a single specific PCR product was verified by melting curve analysis and confirmed on agarose gels.

**Table 2:** Primer pairs for real-time quantitative PCR analysis.

Symbol	Description	Sequence	
GAPD	glyceraldehyde-3-phosphate dehydrogenase	AGCCTCAAGATCATCAGCAATG	(Forward)
		ATGGACTGTGGTCATGAGTCCTT	(Reverse)
CXCL10	chemokine (C-X-C motif) ligand 10	TTCCTGCAAGCCAATTTTGT	(Forward)
		ATGGCCTTCGATTCTGGATT	(Reverse)
ADH1C	alcohol dehydrogenase 1C (class I)	GGCTTGACACCATGATGGCT	(Forward)
		GGAGGTACCCCTACAATGACA	(Reverse)
ADH4	alcohol dehydrogenase 4 (class II)	CTGAAACCATGAAAGCAGCC	(Forward)
		GCCGATTATTAGCTCCTCTGG	(Reverse)
ADH6	alcohol dehydrogenase 6 (class V)	CAATACTGCCAAGGTGACTCC	(Forward)
		GCTCCTGCTGCTTTACAACC	(Reverse)
ALDH2	aldehyde dehydrogenase 2	TGGTTACTTCATCCAGCCCAC	(Forward)
		GCTCTCCCAACAACCTCCTCTAT	(Reverse)
ALDH8A1	aldehyde dehydrogenase 8 family, member A1	TGGGAGTCGCTGGTCTGAT	(Forward)
		CTGGGCTTGGCTATCACAGT	(Reverse)
TLR4	toll-like receptor 4	AATCCCCTGAGGCATTTAGG	(Forward)
		CCCCATCTTCAATTGTCTGG	(Reverse)

Threshold cycles (Ct) for each sample were determined by Bio-rad iQ5 optical system software (Bio-Rad Laboratories). The concentration of mRNA ([mRNA]) is represented by the following equation: [mRNA] = M/E^Ct^, where constant M is an arbitrary threshold, E is the efficiency of PCR, Ct is the threshold cycle. All PCR reactions had efficiencies higher than 1.9, as determined experimentally with 4-times serial diluted samples. The relative mRNA concentration of each target gene was calculated as the mRNA concentration of target gene normalized against that of GAPD, as represented by the following equation:
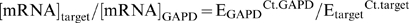



### Western Blot Analysis

Liver biopsies stored in RNAlater were homogenized in PBS, and then boiled for 5 min in SDS-PAGE loading buffer. Samples (4µg total protein each) were separated on 10% SDS PAGE gels. After blotting onto nitrocellulose membranes, ADH1 (cat#sc-137091, Santa Cruz Biotechnology Inc., CA), ADH4 (cat#sc-134249, Santa Cruz Biotechnology Inc.) and β-actin (Clone C4, MP Biomedicals, LLP, Ohio) were probed on separate blots, as all these proteins are of similar sizes. The results were visualized using the SuperSignal West Dura Extended Duration Substrate (Invitrogen) and recorded with an image reader LAS-1000 (Fujifilm).

### Statistical Analysis

All analysis for statistically significant differences was performed with Student's t test with a two-tailed distribution. P values smaller than 0.05 were considered significant.

## Results

### The Integrity of the Microarray Datasets

To compare the global gene expression profiles between NASH livers and normal livers, total RNA samples from both populations (NASH, n = 7; NC, n = 4) were analyzed on CodeLink Human Whole Genome Bioarray. To ascertain the quality of RNA used in microarray analysis, similar datasets of breast cancer tissue generated with the same CodeLink platform were downloaded (GEO accession numbers: GSM144855 to GSM144858) and compared with the liver microarray datasets. As represented by the housekeeping genes listed in Table 3 (upper part), the majority of the genes showed similar expression levels among NASH livers, normal livers and breast cancer tissues. In contrast, drastically elevated expression was detected in liver tissues for the liver-specific genes including apolipoproteins, fatty acid binding protein, hepatic triglyceride lipase, glutathione S-transferase, and alcohol dehydrogenase (Table 3, lower part).

**Table 3:** Comparison of the gene expressions between liver tissues and breast cancer tissues.†

	GenBank Accession #	Gene Description	NASH Liver (n = 7)	Normal Liver (n = 4)	Breast Cancer (n = 4)	NASH Liver/Breast Cancer‡
	NM_000402.2	glucose-6-phosphate dehydrogenase (G6PD)	1.58±0.10§	1.53±0.27	1.37±0.64	1.16
	NM_001101.2	actin, beta (ACTB)	18.19±2.91	16.07±4.88	20.38±19.35	0.89
	NM_000034.2	aldolase A, fructose-bisphosphate (ALDOA)	9.02±1.63	19.02±4.34	6.91±6.18	1.30
	NM_003379.3	villin 2 (ezrin) (VIL2)	1.78±0.42	4.02±1.28	1.38±0.84	1.29
	AL133626.1	H2A histone family, member J	1.66±0.12	1.81±0.22	1.88±0.20	0.88
	NM_002954.3	ribosomal protein S27a (RPS27A)	171.21±46.00	184.38±33.35	91.67±61.77	1.87
	NM_006951.2	TAF5 RNA polymerase II, TATA box binding protein-associated factor	1.64±0.24	1.04±0.25	1.46±0.20	1.13
	NM_001190.1	branched chain aminotransferase precursor (BCATm)	1.32±0.42	1.40±0.64	1.44±0.39	0.92
	NM_000777.2	cytochrome P450, family 3, subfamily A, polypeptide 5 (CYP3A5)	4.91±1.83	8.08±3.40	0.00±0.21	10177.97
	NM_000040.1	apolipoprotein C-III (APOC3)	625.93±126.08	312.83±67.97	0.40±0.00	1579.62
	NM_001443.1	fatty acid binding protein 1, liver (FABP1)	605.55±80.67	250.15±51.55	0.42±0.25	1458.34
	NM_000039.1	apolipoprotein A-I (APOA1)	557.21±58.12	306.33±52.72	0.52±0.16	1071.00
	NM_006744.2	retinol binding protein 4, plasma (RBP4)	606.95±83.36	295.00±43.11	0.68±0.30	888.88
	NM_145740.1	glutathione S-transferase A1 (GSTA1)	312.50±33.64	25.17±14.02	0.42±0.22	742.18
	NM_000667.2	alcohol dehydrogenase 1A (class I), alpha polypeptide (ADH1A)	470.96±40.68	82.50±34.30	0.64±0.11	735.11
	NM_000483.3	apolipoprotein C-II (APOC2)	501.93±30.57	288.10±49.95	1.20±0.75	419.61
	NM_000236.1	hepatic triglyceride lipase (HTGL)	289.35±47.50	142.52±10.06	0.76±0.23	383.05
	NM_000670.2	alcohol dehydrogenase 4 (class II), pi polypeptide (ADH4)	257.14±38.38	6.53±2.17	0.80±0.44	323.14
	NM_000041.1	apolipoprotein E (APOE)	353.83±49.92	258.38±53.30	1.81±0.63	195.99

† Microarray data for breast cancer were downloaded from PubMed, accession #: GSE6304.

‡ Fold difference of gene expression levels (sample mean) between NASH liver tissues and breast cancer tissues.

§ Median normalized gene expression signal as detected by the Codelink microarray. Sample mean ± standard error.

¶ For all the liver specific genes listed, significant differences in gene expression were detected between liver tissues and breast cancer tissues (P<0.05).

### Elevated Expression of Inflammation and Fibrosis Related Genes in NASH Livers

The expression levels of genes involved in the process of inflammation and fibrosis were of immediate attention, as the activities of these genes would provide an ultimate evaluation of the quality of the datasets. Listed in Table 4, most of the chosen housekeeping genes showed similar transcription levels between NASH livers and normal controls (Table 4, upper panel). In contrast, significant differences were observed for many genes known to be involved in inflammation (Table 4, middle panel). These include several members of the HLAs, C-X-C motif containing chemokines and members of the TNF receptor superfamily.

**Table 4:** Comparison of gene expressions between NASH livers and normal controls: house-keeping genes, inflammation and fibrosis related genes.†

	GenBank Accession#	Gene Description	NASH Liver (n = 7)	Normal Liver (n = 4)	NASH/Normal‡	P value§
	NM_000402.2	glucose-6-phosphate dehydrogenase (G6PD)	1.58±0.10	1.53±0.27	1.03	0.875
	NM_001101.2	actin, beta (ACTB)	18.19±2.91	16.07±4.88	1.13	0.723
	NM_000034.2	aldolase A, fructose-bisphosphate (ALDOA), transcript variant 1	9.02±1.63	19.02±4.34	0.47	0.100
	NM_003379.3	villin 2 (ezrin) (VIL2)	1.78±0.42	4.02±1.28	0.44	0.179
	AL133626.1	H2A histone family, member J	1.66±0.12	1.81±0.22	0.91	0.560
	NM_002954.3	ribosomal protein S27a (RPS27A)	171.21±46.00	184.38±33.35	0.93	0.822
	NM_006951.2	TAF5 RNA polymerase II, TATA box binding protein (TBP)-associated factor	1.64±0.24	1.04±0.25	1.59	0.122
	NM_001190.1	branched chain aminotransferase precursor (BCATm)	1.32±0.42	1.40±0.64	0.94	0.922
	NM_002121.4	major histocompatibility complex, class II, DP beta 1 (HLA-DPB1)	70.94±8.30	3.49±1.96	20.31	0.000
	NM_006120.2	major histocompatibility complex, class II, DM alpha (HLA-DMA)	24.91±3.39	4.77±1.41	5.22	0.001
	NM_002121.4	major histocompatibility complex, class II, DP beta 1 (HLA-DPB1)	15.86±2.85	1.01±0.28	15.64	0.002
	NM_002127.3	HLA-G histocompatibility antigen, class I, G (HLA-G)	27.88±2.00	7.97±0.71	3.50	0.000
	NM_019111.2	major histocompatibility complex, class II, DR alpha (HLA-DRA)	153.26±18.42	15.67±4.81	9.78	0.000
	NM_033554.2	major histocompatibility complex, class II, DP alpha 1 (HLA-DPA1)	122.95±15.12	10.77±3.51	11.41	0.000
	X02902.1	mRNA for HLA class II DR-beta 1 (Dw14)	26.29±5.05	2.08±0.74	12.64	0.003
	NM_001565.1	chemokine (C-X-C motif) ligand 10 (CXCL10)	26.04±6.78	1.21±0.47	21.55	0.010
	NM_004887.3	chemokine (C-X-C motif) ligand 14 (CXCL14)	62.97±8.44	3.78±1.79	16.64	0.000
	NM_000609.3	chemokine (C-X-C motif) ligand 12 (stromal cell-derived factor 1) (CXCL12)	13.99±2.63	2.56±1.46	5.47	0.005
	NM_005409.3	chemokine (C-X-C motif) ligand 11 (CXCL11)	0.82±0.10	0.27±0.10	3.08	0.004
	NM_001250.3	tumor necrosis factor receptor superfamily, member 5 (TNFRSF5), transcript variant 1	8.00±1.16	1.57±0.69	5.08	0.001
	NM_000043.3	tumor necrosis factor receptor superfamily, member 6 (TNFRSF6), transcript variant 1	7.22±0.80	2.59±0.76	2.79	0.003
	NM_000072.1	CD36 antigen (collagen type I receptor, thrombospondin receptor) (CD36)	16.55±3.10	2.87±1.63	5.76	0.004
	NM_005505.3	CD36 antigen (collagen type I receptor, thrombospondin receptor)-like 1 (CD36L1)	1.84±0.41	0.76±0.15	2.42	0.041
	NM_030582.2	collagen, type XVIII, alpha 1 (COL18A1), transcript variant 1	29.30±3.83	9.69±2.14	3.02	0.002
	NM_001853.2	collagen, type IX, alpha 3 (COL9A3)	6.00±1.94	1.15±0.66	5.20	0.049
	NM_004369.1	RNA for type VI collagen alpha3 chain	4.73±0.79	1.41±0.73	3.36	0.014
	X52022.1	RNA for type VI collagen alpha3 chain	1.10±0.10	0.28±0.17	3.95	0.011
	NM_001850.3	collagen, type VIII, alpha 1 (COL8A1), transcript variant 1	1.50±0.15	0.43±0.04	3.49	0.000
	NM_015719.2	collagen, type V, alpha 3 (COL5A3)	0.98±0.26	0.17±0.05	5.73	0.019

† The gene expression levels (sample mean ± standard error) shown were median normalized.

‡ Fold difference of gene expression levels (sample mean) between NASH liver tissues and normal liver controls.

§ Two tailed student t test.

The NASH patients included in this study all had certain degrees of fibrosis. It was therefore of great interest to see if the NASH microarray dataset could characterize the disease status. The abnormal deposition of collagen is a structural basis of liver fibrosis. From the datasets, it was noted that several types of collagen and collagen receptor exhibited elevated transcription activity in NASH livers (Table 4, lower panel). The activation of stellate cells from their resting state to myofibroblasts was also suggested by the elevated transcription for genes specific for smooth muscle functions (data not shown).

**Figure 1:**
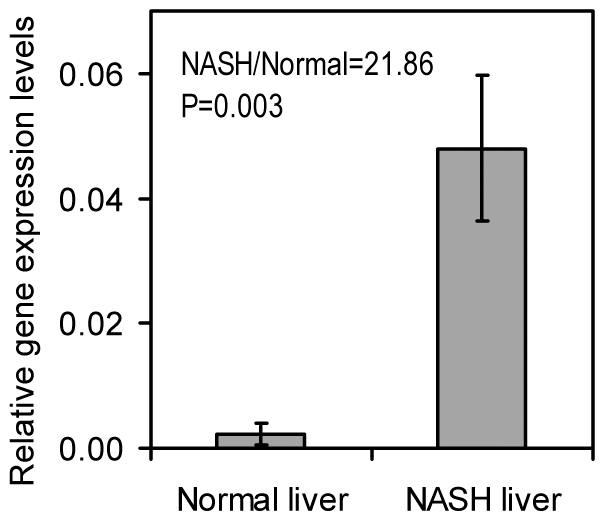
Quantitative RT-PCR analysis of CXCL10 in NASH livers and normal controls (NCs). Quantitative RT-PCR analysis were performed as described in Methods. CXCL10 and GAPD (house-keeping gene) specific primer pairs are specified in Table 2. The complementary DNAs prepared from NASH livers (n = 6) and NCs (n = 6) were analyzed in duplicate. CXCL10 expression level of each sample was normalized with that of GAPD. Sample means of the CXCL10 gene expression levels were plotted with error bars indicating the standard errors.

To confirm the observations made with the microarray technique, cDNA was synthesized from liver RNA and subjected to qRT-PCR analysis (NASH, n = 6; NC, n = 6) with gene specific primer pairs for GAPD and CXCL10 (Table 2). CXCL10 was chosen because it was the most highly activated inflammatory gene based on the microarray datasets. The real-time PCR result (Figure 1) indicated that the transcription level of CXCL10 in NASH liver was 21.86 times of that in normal healthy liver (P = 0.003), which is consistent with the microarray data (in which, NASH/normal = 21.55).

Overall, the elevated transcription of inflammation and fibrosis related genes validated both the NASH and normal control samples for further investigation on the gene expression patterns of NASH livers.

### Elevated Expression of Genes Responsible for Alcohol Catabolism

When comparing the microarray datasets between NASH livers and normal livers, alcohol dehydrogenase 4 (ADH4) stands out as even more elevated than the inflammation related genes with an almost 40 fold increase in NASH livers (Table 5, upper panel). This observation prompted us to examine other genes in the ADH family. Of the 7 well established members of ADH family, ADH1A, ADH1B, ADH1C, ADH4, ADH5 and ADH6 showed significantly elevated transcription activity in NASH livers compared with normal livers (Table 5, upper panel). ADH7 also seemed to be highly elevated in NASH livers. However, the absolute signal for ADH7 gene transcription is low in both liver samples and the difference is statistically insignificant (P>0.05). This is consistent with the fact that ADH7 is usually expressed in stomach; less in liver [14].

**Table 5:** Comparison of gene expressions between NASH livers and normal controls: alcohol metabolism and TLR4 related genes.†

	GenBank Accession#	Gene Description	NASH Liver (n = 7)	Normal Liver (n = 4)	NASH/Normal‡	P value§
	NM_000667.2	alcohol dehydrogenase 1A (class I), alpha polypeptide (ADH1A)	470.96±40.68	82.50±34.30	5.71	0.000
	NM_000668.3	alcohol dehydrogenase IB (class I), beta polypeptide (ADH1B)	95.00±21.68	34.23±9.89	2.78	0.034
	NM_000669.2	alcohol dehydrogenase 1C (class I), gamma polypeptide (ADH1C)	76.58±16.72	18.75±5.60	4.08	0.013
	NM_000670.2	alcohol dehydrogenase 4 (class II), pi polypeptide (ADH4)	257.14±38.38	6.53±2.17	39.40	0.001
	NM_000671.2	alcohol dehydrogenase 5 (class III), chi polypeptide (ADH5)	10.95±1.97	2.71±1.16	4.04	0.006
	NM_000672.2	alcohol dehydrogenase 6 (class V) (ADH6)	36.68±5.09	3.56±1.14	10.30	0.000
	NM_000673.2	alcohol dehydrogenase 7 (class IV), mu or sigma polypeptide (ADH7)	0.34±0.08	0.01±0.11	26.96	0.053
	NM_000773.2	cytochrome P450, family 2, subfamily E, polypeptide 1 (CYP2E1)	423.85±42.84	194.97±50.22	2.17	0.010
	NM_001752.1	catalase (CAT)	12.27±2.16	0.97±0.39	12.70	0.002
	NM_000689.3	aldehyde dehydrogenase 1 family, member A1 (ALDH1A1)	45.47±9.70	5.61±2.18	8.11	0.006
	NM_003888.2	aldehyde dehydrogenase 1 family, member A2 (ALDH1A2)	0.70±0.13	0.27±0.09	2.57	0.023
	NM_000692.3	aldehyde dehydrogenase 1 family, member B1 (ALDH1B1)	63.79±5.52	15.01±2.81	4.25	0.000
	NM_000690.2	aldehyde dehydrogenase 2 family (mitochondrial) (ALDH2)	4.47±1.07	0.71±0.24	6.30	0.012
	NM_000695.2	aldehyde dehydrogenase 3 family, member B2 (ALDH3B2)	0.37±0.10	0.24±0.09	1.55	0.354
	NM_003748.2	aldehyde dehydrogenase 4 family, member A1 (ALDH4A1)	25.86±3.87	16.25±2.08	1.59	0.058
	NM_001080.3	aldehyde dehydrogenase 5 family, member A1 (ALDH5A1)	3.79±0.41	1.31±0.45	2.89	0.004
	NM_000693.1	aldehyde dehydrogenase 6 mRNA, complete cds	0.49±0.12	0.33±0.08	1.48	0.280
	NM_000694.1	aldehyde dehydrogenase ALDH7	8.17±0.52	3.26±0.21	2.51	0.000
	NM_022568.2	aldehyde dehydrogenase 8 family, member A1 (ALDH8A1)	24.43±1.84	5.46±0.57	4.47	0.000
	NM_000696.2	aldehyde dehydrogenase 9 family, member A1 (ALDH9A1)	89.27±11.55	75.57±16.01	1.18	0.513
	NM_003266.2	toll-like receptor 4 (TLR4), transcript variant 3	1.34±0.12	0.99±0.08	1.35	0.034
	NM_000591.1	CD14 antigen (CD14)	17.47±5.34	14.85±6.85	1.18	0.772
	NM_004139.2	lipopolysaccharide binding protein (LBP)	66.88±11.71	167.76±15.77	0.40	0.002
	NM_015364.2	MD-2, lymphocyte antigen 96 (LY96)	5.21±0.97	4.24±2.77	1.23	0.758

† The gene expression levels (sample mean ± standard error) shown were median normalized.

‡ Fold difference of gene expression levels (sample mean) between NASH liver tissues and normal liver controls.

§ Two tailed student t test.

Besides ADH, the liver has two additional enzymatic systems to remove alcohol: microsomal ethanol oxidizing system (MEOS) in endoplasmic reticulum and catalase in the peroxisomes. Significant elevation in gene transcription was observed for catalase and cytochrome P450 2E1 (CYP2E1), the major functional component of MEOS. Together with the augmented transcriptional activities of ADH genes, these results indicated a high demand for removing alcohols from the circulations of NASH patients.

The immediate products from the aforementioned alcohol oxidizing systems are aldehydes, which are more toxic than alcohol and therefore need to be removed immediately, usually by aldehyde dehydrogenases (ALDHs). The fact that there are many isoforms of ALDH encoded in human genome reflects the importance of these genes. Except ALDH3, 4, 6 and 9, all other isoforms of ALDH were found to be transcribed significantly higher in NASH livers compared to normal livers (Table 5, middle panel).

**Figure 2:**
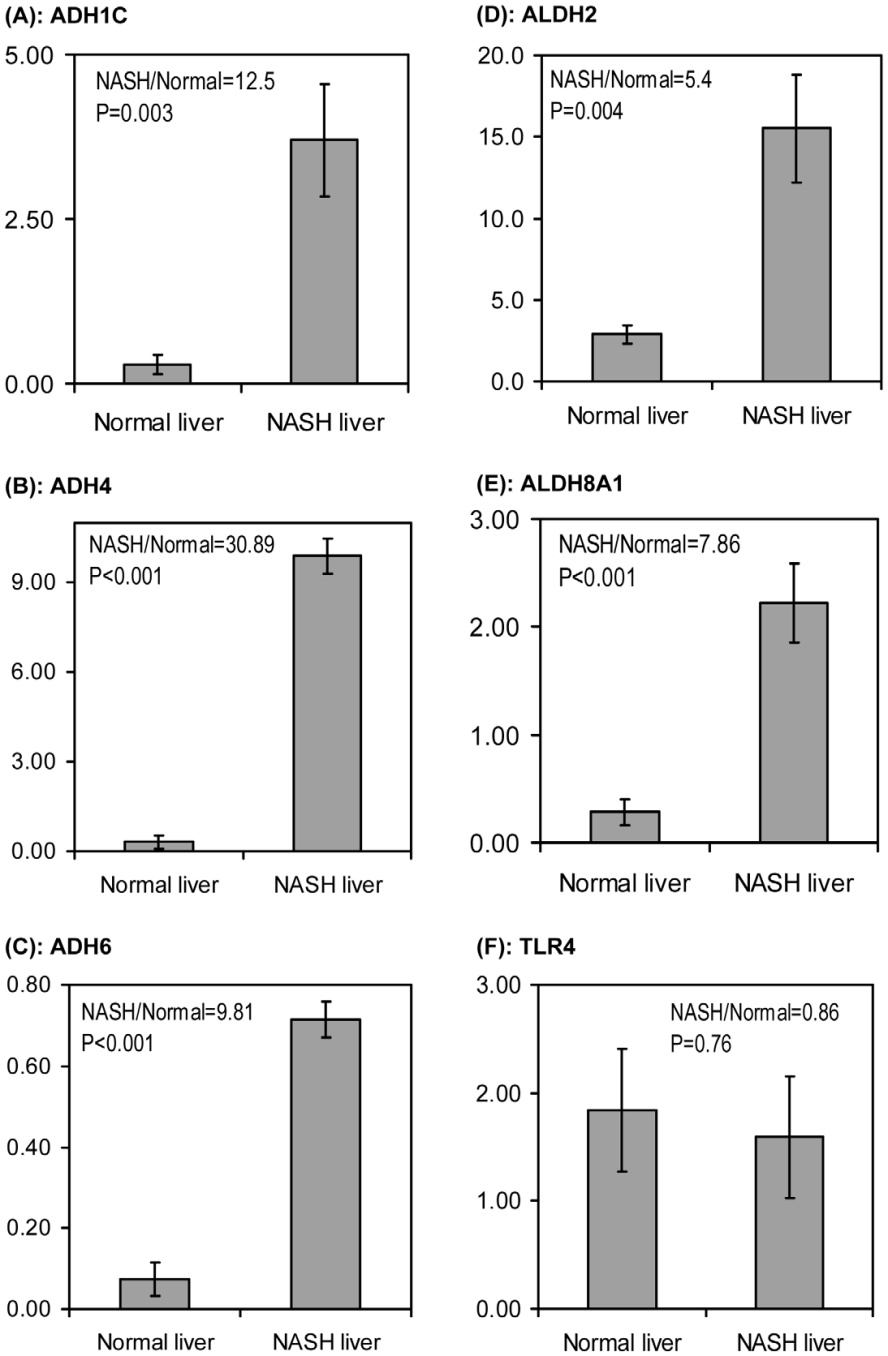
Quantitative RT-PCR analysis with NASH livers and NCs. Gene expression levels of (A) ADH1C, (B) ADH4, (C) ADH6, (D) ALDH2, (E) ALDH8A1 and (F) TLR4 were examined by qRT-PCR as described in Methods. The complementary DNAs prepared from NASH livers (n = 6) and NCs (n = 6) were analyzed in duplicate. PCR primer pairs are specified in Table 2. Gene expression levels of each sample were normalized with those of GAPD. Sample means were plotted with error bars indicating the standard errors.

Since none of the patients had any history of alcohol consumption, these results were considered extraordinary and therefore real-time PCR was performed to confirm the microarray data. As ADH is the major mechanism for alcohol metabolism, several ADH genes were targeted. Again, with GAPD as internal reference, these ADH genes showed greater transcriptional activity in NASH livers than in normal livers (Figure 2, A, B and C). In NASH livers, the ADH1C transcription was 12.5 times that of normal livers. For ADH4 and ADH6, it was 30.89 and 9.81 times, respectively. Note that these numbers showed a similar pattern given by microarray data, in which NASH/normal = 4.08 (ADH1C), 39.40 (ADH4), and 10.30 (ADH6). Significantly higher transcriptional activities of ALDH2 and ALDH8A1 were also observed by qRT-PCR in NASH livers compared to normal livers (Figure 2, D and E), confirming the previously described microarray data.

### NASH Liver Did Not Exhibit Elevated Gene Transcription for TLR4 and Related Genes

Studies with rodent models suggested that TLR4 mediated pathway is elevated in NASH liver[15], [16]. From our microarray data, the gene transcription of TLR4 was slightly elevated in NASH patients (NASH/normal = 1.35, P<0.05). Yet the transcription of TLR4 related genes CD14, LBP and MD-2 were not elevated (Table 5, lower panel). In fact, a significant decrease in LBP transcription was detected with these patients (NASH/normal = 0.4, P = 0.002). Real-time PCR analysis with an overlapping but different subgroup of NASH samples indicated that TLR4 gene transcription was not elevated in NASH livers (Figure 2F). Therefore, the small elevation in TLR4 gene transcription observed with microarray analysis may not be a universal phenomenon in NASH liver.

### Elevated Protein Expression of ADH1 and ADH4 in NASH Livers

**Figure 3:**
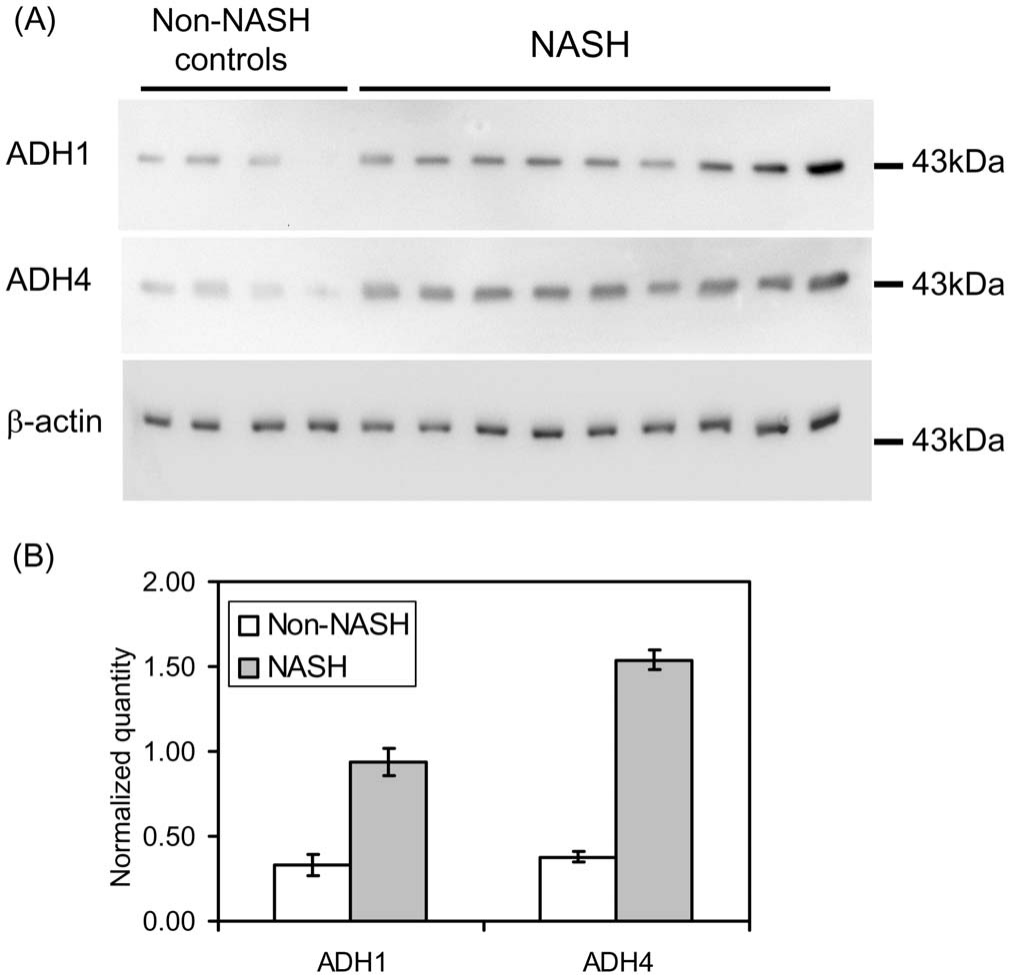
Elevated expression of ADH1 and ADH4 proteins in NASH livers. (A) Western blot analyses were performed with lysates prepared from NASH livers and NCs. The NCs were of normal BMI and free from steatosis. Separate blots were probed for ADH1, ADH4 and β-actin as these proteins all migrate at about 43 kDa. While similar signals for actin were detected for all samples, the NASH livers exhibited stronger signals for both ADH1 and ADH4. (B) The Western blot results were quantitated with NIHimage software. The normalized quantities of the ADH1 and ADH4 signals were the densities of the ADH bands divided by that of the β-actin bands, respectively. The mean values for NASH and NC were plotted with error bars representing the standard errors of the means.

To examine the expression of ADH at protein level, Western blot analyses were performed with the lysates made from NASH liver biopsies (NASH, n = 9; Control, n = 4). As normal healthy liver tissue from adolescent is not available, non-NASH liver biopsies free from steatosis were used as controls. Blots were probed with antibodies specific for ADH1 and ADH4, respectively. Separate blot was also probed for β-actin as loading controls. While all samples had similar signals for actin, NASH patients exhibited higher expression of ADH1 and ADH4 (Figure 3A). Quantitation of the results with NIHimage software indicated that there were significant increases in ADH1 and ADH4 proteins in NASH livers: for ADH1, NASH/normal control = 2.8, P<0.001; for ADH4, NASH/normal control = 4.1, P<0.001 (Figure 3B).

## Discussion

### Liver Specific Genes, Inflammation and Fibrosis Related Genes

Gene transcription of NASH livers was examined by a whole genome DNA microarray and qRT-PCR techniques. Although the sample size in the microarray analysis (NASH, n = 7; NC, n = 4) is relatively small, convincing results were observed with a larger sample size (NASH, n = 6; NC, n = 6) in qRT-PCR analysis. The similar gene expression patterns detected by both techniques testify to the reliability of the techniques and the results. We acknowledge that the clinical information of the normal liver RNA purchased from Admet is limited. However, we are confident that these samples are appropriate to serve as controls, not only because Admet guaranteed that these samples were derived from normal healthy livers intended for transplantation, but also for the following reasons: 1) each of the normal controls had a moderate BMI, therefore they were not likely to have NASH (Table 1); 2) comparison with similar gene transcription datasets of human breast cancer showed that both the normal control RNA and NASH RNA exhibited similar good quality, that is, liver specific genes were highly expressed in both groups of liver tissue (Table 3); and 3) these normal liver samples showed lower transcription of inflammation and fibrosis related genes as compared to NASH liver samples, in the context of similar gene transcription for housekeeping genes between these two groups (Table 4 and Figure 1).

### Genes Responsible for Alcohol Catabolism

Alcohol is promptly removed from circulation via three cellular mechanisms mainly found in liver: alcohol dehydrogenases in the cytosol; MEOS (with cytochrome P450 2E1 as the major component) in endoplasmic reticulum and catalase in peroxisomes. All known ADH genes except ADH7, together with cytochrome P450 2E1 and catalase genes, showed elevated transcription in NASH livers. The different behavior of ADH7 from other alcohol oxidizing enzymes is predicted by the fact that ADH7 is not a liver enzyme [17]. The elevated expression for ADH1 and ADH4 at protein level were also observed in NASH livers (NASH, n = 9; NC, n = 4), consistent with the elevated mRNA levels for these genes. The significant elevation of the activity of these enzymes indicated that 1), alcohol concentration was elevated in the circulation and 2), it is a high priority for NASH patients to remove alcohol from the circulation.

All our adolescent patients claimed that they were not regular drinkers of alcoholic beverages. We also were assured by their parents that these adolescent patients had no access to alcoholic beverages. Even, in the event that some of our patients did ingest alcohol, it was certainly not consistent long-term alcohol use. In considering other possible sources of alcohol (aspartame, fruit), we also did a three day food record before any biopsy was performed. We found no significant sources for alcohol.

Where did the alcohol come from? For people who do not have access to alcoholic beverages, circulating alcohol mainly comes from diet and intestinal bacteria. For instance, the concentration of methanol in the human body could increase by an order of magnitude after consumption of fruit [18]. Alcohol production by intestinal bacteria was observed long ago in rodents and humans [19], [20]. Studies with obese ob/ob mice indicated that intestinal bacteria are the largest source of circulating alcohol in obese animals [10]. When intestinal bacterial growth was inhibited with neomycin, a 50% decrease of circulating alcohol was observed in obese mice. Based on this observation, Diehl and her colleagues hypothesized that intestinal production of alcohol may contribute to the genesis of non-alcoholic liver diseases [10]. Coincidently, our microarray data provided molecular biological evidence in support of this hypothesis. To pursue this avenue, the immediate future directions are 1) to examine the enzymatic activities of the enzymes related to alcohol catabolism, and 2) to measure the concentrations of circulating alcohol in NASH patients and healthy subjects. If these studies come to the same conclusion as the mRNA data suggest, oral antibiotics, lactobacillus or other means of altering colonic bacteria might be a treatment for NASH.

Our results are also consistent with two interesting discoveries. First, it was observed that cytochrome P450 2E1 is ethanol inducible, with a 4–10 fold increase in liver biopsies of recently drinking subjects [21]. Second, increased P450 2E1 was detected in NASH patients [22].

### Alcohol and Steatosis

The pathomechanisms of alcohol in liver disease have been intensively studied in alcoholic liver disease. It has long been observed that ethanol stimulates hepatic fatty acid synthesis [23]. The oxidation of alcohol to aldehyde by ADH concomitantly reduces NAD to NADH. The increased NADH promotes fatty acid synthesis and opposes lipid catabolism, leading to fat accumulation in hepatocytes [23], [24]. Elevated ADH level as suggested by our microarray data strengthens the connection between alcohol metabolism and steatosis.

### Alcohol and Liver Inflammation

One possible mechanism for alcohol induced liver inflammation begins with mucosal injury in the upper gastrointestinal tract induced by alcohol. This leads to increased permeability for macromolecules like LPS endotoxin. The resulting endotoxemia would then activate TLR4 mediated signaling and culminate in the release of pro-inflammatory cytokines (reviewed in [25]). If the intestinal bacteria could cause elevated production of alcohol, it is conceivable that the NASH patients may suffer from endotoxemia. However, for all the TLR4 related genes examined (TLR4, LBP, CD14, and MD-2), the microarray and real-time PCR analysis did not show significant activation at the mRNA level in NASH patients. Although more convincing conclusion awaits studies of TLR4 signaling at the protein level in NASH patients, our data suggest an emphasis on direct toxicity of alcohol per se.

Szabo and her colleagues reported that alcohol directly suppressed the NF-κB activity in monocytes [26]. Their results are in concert with a previous report that ethanol suppressed TNF activity [27]. However, McClain's group reported the opposite. They found that peripheral blood monocytes from patients who had alcoholic hepatitis have spontaneous TNF production and enhanced LPS-stimulated TNF production [28]. Their finding was confirmed with an in vivo experiment using rats chronically fed alcohol [29]. Work in Nagy's lab further revealed that ERK1/2 and Egr-1 are important mediators in elevated TNF-α production after chronic ethanol exposure [30], [31]. The reality of the ethanol effect on TNF-α production was made clear by Kolls' group. In a time-series alcohol experiment, they demonstrated that acute ethanol exposure suppressed TNF production, while long-term treatment significantly up-regulated TNF production [32]. More importantly, the increase in TNF production was associated with increased generation of reactive oxygen species (ROS).

Therefore, ROS could be the important mediator between alcohol and TNF. Alcohol is known to induce cytochrome P450, mainly CYP2E1 [33]. Metabolism by CYP2E1 generates superoxide and other free radicals collectively known as ROS. There are two possible mechanisms for ROS to induce TNF production. Firstly, ROS could serve as signaling molecules to activate NF-κB, leading to elevated transcription of TNF-α [34]; Secondly, ROS could activate TNF-α converting enzyme, the enzyme responsible for the processing of the transmembrane TNF-α precursor [35].

**Figure 4:**
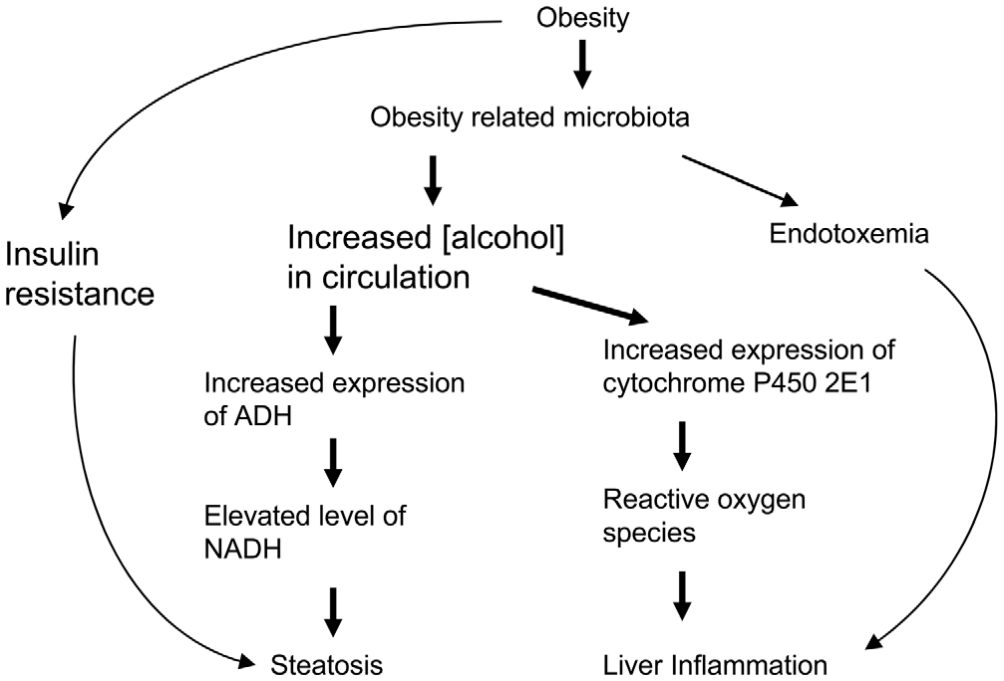
The alcohol hypothesis of non-alcoholic liver diseases. Non-alcoholic fatty liver diseases are commonly associated with obesity. In addition to the well-known mechanisms that obesity leads to steatosis via insulin resistance, and obesity related bacteria facilitate liver inflammation, the gene transcription data reported here support a central role for alcohol in the pathogenesis of NAFLD. Overgrowth of alcohol-producing bacteria in the intestine of obese patients likely causes increased alcohol in the circulation, which in turn induced the expression of genes for alcohol catabolism, including ADH and cytochrome P450 2E1. The increased activity of ADH results in the elevated level of NADH, which favors fatty acid synthesis and opposes its break down, leading to steatosis; the elevated P450 2E1 could generate excessive ROS, a known cause for liver inflammation.

In summary, the gene expression studies of NASH livers revealed elevated transcription of enzymes in ALL the alcohol metabolism pathways: ADH pathway, catalase pathway and the MEOS pathway. Elevated expression of ADH1 and ADH4 at protein level were also observed in NASH livers. These results support a central role for alcohol in the development of NASH in obese patients, as summarized in Figure 4 and for the first time, provide molecular evidence in support of Diehl et al.'s hypothesis that alcohol is an important mediator for the development of non-alcoholic fatty liver diseases [10]. Our findings suggest a mechanism that offers a single explanation for the similar histological findings in alcoholic liver disease and NASH [3]. Our findings might provide a basis for studies on the prevention and treatment of NASH.
